# Development and In Vitro Analysis of Layer-by-Layer Assembled Membranes for Potential Wound Dressing: Electrospun Curcumin/Gelatin as Middle Layer and Gentamicin/Polyvinyl Alcohol as Outer Layers

**DOI:** 10.3390/membranes13060564

**Published:** 2023-05-30

**Authors:** Ssu-Meng Huang, Shih-Ming Liu, Hua-Yi Tseng, Wen-Cheng Chen

**Affiliations:** 1 Advanced Medical Devices and Composites Laboratory, Department of Fiber and Composite Materials, Feng Chia University, Taichung 407, Taiwan; dream161619192020@gmail.com (S.-M.H.); 0203home@gmail.com (S.-M.L.); huahuayi98@gmail.com (H.-Y.T.); 2 Department of Fragrance and Cosmetic Science, College of Pharmacy, Kaohsiung Medical University, Kaohsiung 807, Taiwan; 3 Dental Medical Devices and Materials Research Center, College of Dental Medicine, Kaohsiung Medical University, Kaohsiung 807, Taiwan

**Keywords:** electrospun, nanofiber, layer-by-layer, membranes, drug release, antibacterial, cytotoxicity, wound dressing

## Abstract

Nanofibrous membranes made of hydrogels have high specific surface areas and are suitable as drug carriers. Multilayer membranes fabricated by continuous electrospinning could delay drug release by increasing diffusion pathways, which is beneficial for long-term wound care. In this experiment, polyvinyl alcohol (PVA) and gelatin were used as membrane substrates, and a sandwich PVA/gelatin/PVA structure of layer-by-layer membranes was prepared by electrospinning under different drug loading concentrations and spinning times. The outer layers on both sides were citric-acid-crosslinked PVA membranes loaded with gentamicin as an electrospinning solution, and the middle layer was a curcumin-loaded gelatin membrane for the study of release behavior, antibacterial activity, and biocompatibility. According to the in vitro release results, the multilayer membrane could release curcumin slowly; the release amount was about 55% less than that of the single layer within 4 days. Most of the prepared membranes showed no significant degradation during immersion, and the phosphonate-buffered saline absorption rate of the multilayer membrane was about five to six times its weight. The results of the antibacterial test showed that the multilayer membrane loaded with gentamicin had a good inhibitory effect on *Staphylococcus aureus* and *Escherichia coli*. In addition, the layer-by-layer assembled membrane was non-cytotoxic but detrimental to cell attachment at all gentamicin-carrying concentrations. This feature could be used as a wound dressing to reduce secondary damage to the wound when changing the dressing. This multilayer wound dressing could be applied to wounds in the future to reduce the risk of bacterial infection and help wounds heal.

## 1. Introduction

Acute and chronic wounds place heavy burdens on patients and healthcare systems around the world; research on wound dressings that could effectively treat wounds in a short period is a reliable method to reduce the burden [1]. Based on appearance, wound dressings can be divided into hydrogels, membranes, sponges, foams, and nanofibers [2]. Among them, the porous structure of nanofibers made by electrospinning could promote the absorption of wound tissue fluid and allow gas circulation to prevent bacterial invasion. As the electrospun structure is similar to the extracellular matrix of the skin, it is conducive to cell attachment, migration, growth and differentiation, and angiogenesis [3,4]. In addition, nanofibrous membranes prepared by hydrogel electrospinning have been used in drug delivery systems due to their excellent drug loading and drug release efficiency [5]. Optimal drug release rates could be achieved by designing nanofibers through osmotic mixing parameters [6], coaxial fibers [7], co-spinning, microemulsion methods [8], auxiliary carriers, and surface loading. In the literature, multilayer membranes produced by continuous electrospinning lead to diffusion paths extending from the middle layer to the outer layer [9]. The release of drugs in the middle layer could be controlled to realize the possibility of separating and releasing drugs with different functions at different times. Depending on the application, drugs are loaded onto different layers of the nanofibrous layer, making the application of the composite membranes more versatile.

Traditional wound dressings only cover the wound and keep bacteria out. They are powerless to prevent wound infection and promote tissue healing. A multifunctional wound dressing that aids in wound repair by adding antimicrobial or healing-promoting drugs must be developed. Therefore, the choice of drug carrier plays a crucial role. In this study, a three-layer nanofiber laminated sandwich structure membrane was prepared by continuous electrospinning, and the drug in the hydrogel was loaded on the membrane as the electrospinning solution to develop a multifunctional wound dressing. The materials used to make the electrospun layers included polyvinyl alcohol (PVA) hydrogel, cross-linker of citric acid (CA), gentamicin antibiotic, gelatin hydrogel, and curcumin. PVA is a hydrophilic hydrogel with good biocompatibility and biodegradability; it is commonly used in the biomedical field [10]. However, PVA is easily degraded when exposed to water, so its structural stability often requires improvement by crosslinking. CA is a naturally generated organic acid with the advantages of nontoxicity, easy access, and biodegradability [11]. CA has the ability to crosslink PVA and improve water stability through esterification [12]. Gentamicin is an aminoglycoside antibiotic that is effective against Gram-positive and -negative bacteria. Its antibacterial mechanism combines with bacterial ribosomes to affect the synthesis of bacterial proteins, thereby destroying the integrity of bacterial cell membranes to achieve antibacterial effects [13]. Gelatin is made from collagen in animal bones, tendons, and skin through acid or alkali hydrolysis. It has good biocompatibility and biodegradability, so it is often used in drug carriers, tissue engineering, and the food industry [14,15]. Curcumin is a natural polyphenolic compound extracted from the rhizome of turmeric [16]. It has anticancer, antioxidant, anti-inflammatory, and antibacterial properties, and it has been used for cancer, cardiovascular disease, diabetes, and arthritis [17].

This study mainly aimed to prepare a multilayer membrane with a sandwich structure capable of absorbing wound exudate and having antibacterial properties, slow drug release, and good biocompatibility. It would provide a favorable environment for wound healing and release drugs to accelerate such healing. In this study, PVA and gelatin were used as electrospun hydrogels to prepare multilayer membranes with hydrophilic, antibacterial, and sustained drug-release properties by continuous electrospinning. The middle layer was a curcumin-loaded gelatin membrane, and the outer layer was a post-crosslinked PVA membrane loaded with the gentamicin antibiotic layer by layer to form a sandwich structure. The physical properties, bacteriostatic properties, drug release capability, and biocompatibility of the multilayer membranes under different drug concentrations and electrospinning time composite parameters were investigated.

## 2. Materials and Methods

### 2.1. Materials

The PVA used (Mw: 200,000–240,000 g/mole, Beijing Guoren Yikang Technology Co., Ltd., Beijing, China) was a white-to-beige powder, with a molecular formula of (C_2_H_4_O)n, a density of 1.3 g/cm^3^, and a melting point of 230–240 °C. Other materials included pure pharmaceutical-grade CA 1-hydrate (Panreac Química, SA, Barcelona, Spain), gelatin (type B, average molar mass of 40,000–50,000 g/mole, Sigma–Aldrich, St. Louis, MO, USA), gentamycin (Siu Guan Chemical Industry Co., Ltd., Chiayi, Taiwan), and curcumin from *Curcuma longa* (turmeric) powder (Sigma–Aldrich, St. Louis, MO, USA).

### 2.2. Preparation of Middle Layer, Outer Layer, and Multilayer Membrane

#### 2.2.1. Middle Layer Preparation

A 30 wt.% gelatin solution was prepared by adding 4.37 g of gelatin to 10 mL of 40 vol.% acetic acids at 60 °C and stirring at 300 rpm for 6 h. Then, 0.23, 0.35, and 0.48 g of curcumin were added and stirred for 2 h to form 5 wt.%, 7.5 wt.%, and 10 wt.% of G_C5, G_C7.5, and G_C10 solutions, respectively (Table 1). Electrospinning was performed on the prepared solution, the distance between the needle tip and the collecting plate was 8 cm, the liquid feeding rate was 0.5 mL/h, and the voltage parameter was 16 kV. The middle layer was then prepared, and the optimal curcumin sample was selected in accordance with the spinning situation and surface structure.

#### 2.2.2. Outer Layer Preparation

According to previous experimental results [18], when the CA concentration is 20 wt.%, the crosslinked PVA membrane does not degrade in the solution and maintains a good fiber shape. Therefore, CA20 was selected as the optimal concentration of CA-crosslinked PVA. The optimal condition for CA20 was used, and gentamicin was added for the subsequent electrospinning experiments.

A total of 0.87 g PVA was added to 10 mL of double distilled water (ddH_2_O) at 100 °C and vigorously stirred to form an 8 wt.% PVA solution. Then, 20 wt.% CA relative to PVA and 1 mL gentamicin at concentrations of 5, 10, 20, and 40 mg/10 mL were added to the solution and stirred at room temperature to prepare the corresponding CA20_G5, CA20_G10, CA20_G20, and CA20_G40 solutions for electrospinning, respectively (Table 2). The needle tip was 14 cm away from the collecting plate, the flow rate was 0.5 mL/h, and the voltage was 15 kV. The mentioned fiber membrane was subjected to the second crosslinking under heat at 150 °C for 1 h. Then, after the surface morphology and structural composition of the sample were observed, antibacterial and cytotoxicity tests were carried out to select the optimal concentration parameters.

#### 2.2.3. Sandwich Structure Preparation

After the optimal parameters of the middle layer and the outer layer solution were selected, the electrospinning time of the middle layer was fixed, whereas the spinning time of the outer layer differed for the preparation of a sandwich structural multilayer membrane. First, the G_C5 group with the best middle layer was used for spinning. The middle layer parameters were as follows: spinning flow rate of 0.5 mL/h, working distance of 8 cm, voltage of 16 kV, and collection of 1 h. Then, the CA20_G40 group of the outer layer was selected for electrospinning on both sides of the middle layer membrane. The outer layer parameters were as follows: electrospinning flow rate of 0.5 mL/h, working distance of 14 cm, and voltage of 15 kV. The double-sided spinning times on each side were separately set to 1, 2, 3, and 4 h. Then, the designated sandwich structure of PVA-gelatin-PVA was subjected to thermal crosslinking under heat at 150 °C for 1 h to obtain P1G1, P2G1, P3G1, and P4G1 groups (Table 3). The verifications of different process conditions were compared in terms of physicochemical properties, drug release, antibacterial activity, and biocompatibility.

### 2.3. Physicochemical Analysis of Multilayer Membranes

#### 2.3.1. Microstructural Observation by Optical Microscopy (OM)

OM analysis (CK, Olympus, Tokyo, Japan) was applied to initially observe whether electrospun droplets/beads were formed in continuous fibers. In addition, cytotoxicity characterization was observed by OM.

#### 2.3.2. Scanning Electron Microscopy (SEM)

Samples were cut into squares of 0.5 × 0.5 cm and plated with metal, and the fibers and fracture patterns on the multilayer membranes were observed by SEM (S-3400N, Hitachi, Tokyo, Japan).

#### 2.3.3. Fourier Transform Infrared Spectroscopy–Attenuated Total Reflectance (FTIR–ATR)

FTIR (Nicolet iS5, Thermo Fisher Scientific, Waltham, MA, USA) spectra were tested by ATR to confirm the functional groups and resulting changes to determine whether the additives were chemically bonded to the polymer.

#### 2.3.4. Tensile Strength Testing

After the thickness was measured, the samples were cut into dumbbell-shaped test pieces following ASTM D882 and subjected to tensile testing by a universal testing machine (HT-2402, Hung Ta, Taichung, Taiwan) at a tensile rate of 2 mm/min until the test piece broke. Then, the tensile strength was recorded.

#### 2.3.5. Water Absorption

A 0.5 × 0.5 cm square sample membrane after mass measurements was immersed in 5 mL of phosphate-buffered saline (PBS; Gibco, Thermo Fisher Scientific Inc., Waltham, MA, USA) and kept at 37 °C during the measurement. Samples were measured at set time points. Excess water from the surface was then wiped off with a saturated sponge, and the wet membrane was weighed to obtain the change. The sample water absorption formula is as follows [19]:

$$Water\;absorption\left(mL/g\right)=\frac{W_{x}-W_{o}}{W_{o}}$$

Among them, W*_x_* and W*_o_* in the formula are the weighted mass of the soaked membrane at a specific time, namely 1, 2, 4, and 8 h and 1, 2, and 4 days, and the initial weight of the membrane, respectively.

#### 2.3.6. Drug Release In Vitro

The 1.0 × 1.0 cm square samples were immersed in 2 mL of PBS and tested at 37 °C for 1, 2, 4, 8, 24, 48, 72, and 96 h. All fluid was sequentially removed at set time points and then supplemented with 2 mL of PBS. The liquid taken out at each time point was mixed with 99.8% ethanol at a volume concentration of 1:1, and the absorbance was measured under OD_435_ nm ultraviolet–visible (UV–vis) light. The measured absorbance was then substituted into the calibration line of curcumin to calculate the released concentration of curcumin.

### 2.4. Antibacterial Activity

The antibacterial qualitative test adopted the agar diffusion test method, and trypsin soybean broth was used as the medium. The sample was cut into a circle with a diameter of 6 mm, sterilized with UV light for 24 h, placed on a sterile plate, and incubated at 37 °C for 24 h. The inhibition zone was determined to judge its antibacterial effect. The antibacterial activity was quantitatively determined via the broth dilution method. First, 0.001 g of the sample was soaked in 1 mL of bacterial suspension with an OD_595_ nm of 0.2 and incubated at 37 °C. The tests were divided into a blank group, a sterilization control group, and an experimental group. After culturing was performed for 24 h, 100 μL of the bacterial liquid was taken out, and the OD_595_ value was measured with an ELISA microplate reader (SPECTROstar Nano, BMG LABTECH, Offenburg, Germany).

### 2.5. Biocompatibility In Vitro

#### 2.5.1. Cytotoxicity

The L929 cell line selected for the cytotoxicity test was mouse fibroblasts, and the test was carried out per the specifications of ISO 10993-5 to evaluate the in vitro cytotoxicity of the drug. The media followed the L929 culture recommendations. Quantitative testing of cytotoxicity started with the preparation of sample extracts. Considering that the thickness of the membrane sample was less than 0.5 mm, it was soaked in a medium at a ratio of 6 cm^2^/1 mL to prepare the sample extracts. The experimental groups included (1) a blank group, which simulated the normal cell culture process; (2) a positive control group mixed with 15% dimethylsulfoxide (DMSO); (3) a negative control group, which used high-density polyethylene (HDPE).

A total of 100 µL of the medium and L929 cells with a cell concentration of 1 × 10^4^ cells/well were added into a 96-well microtiter plate and incubated at 37 °C in a 5% CO_2_ incubator for 24 h. Then, the medium was taken out, the extract was added, and the mixture was incubated for 24 h. Next, 100 μL/well of new cell culture medium and 50 μL/well of XTT cell proliferation assay kit were added and mixed well, placed in a 37 °C incubator, and reacted with 5% CO_2_ for 4 h to prolong the reaction. Then, ELISA was used to measure the absorbance at OD_492_ nm, and the absorbance by the XTT method was directly proportional to the number of active cells.

The preparation of the sample extract and control group for the qualitative test of cytotoxicity was the same as that of the quantitative cytotoxicity test. The culture medium was 1000 µL, and L929 cells with a cell concentration of 1 × 10^5^ cells/well were used as the substrate and placed into a 48-well microliter plate at 37 °C and 5% CO_2_. After 24 h in an incubator, the cell types were observed under an inverted microscope.

#### 2.5.2. Fibroblast L929 Cell Proliferation and Attachment

After the sample was cut into 1 × 1 cm^2^, L929 cells were inoculated on the sample at a concentration of 1 × 10^5^ cell/well; placed in a 48-well culture plate; cultured for 1, 2, 3, 4, and 5 days; and mixed with Alamar blue proliferation assay kit for 4 h of reaction. Cell proliferation was then detected at OD_570_ and OD_595_ nm with an absorbance ELISA reader.

Attachment status change was observed after culturing for 1 h, 1 day, and 2 days, followed by washing with PBS and dehydration and fixation with 2.5% glutaraldehyde, paraformaldehyde, and alcohol of different concentrations in turn. Then, the cells were metal plated on the test piece. After culturing for 1 h, 1 day, and 2 days, the changes in the adherent state of the L929 cells were observed. Then, the cells were washed with PBS, dehydrated, fixed with 2.5% glutaraldehyde, paraformaldehyde, and different concentrations of alcohol in turn, and plated in metal plates for SEM observation.

### 2.6. Statistical Analysis

In this experiment, IBM SPSS Statistics 20 was used to investigate the tension strength and cell proliferation, and the two-sample *t*-test and ANOVA were used for statistical analysis.

## 3. Results

### 3.1. Middle Layer Selection

**Figure 1:**
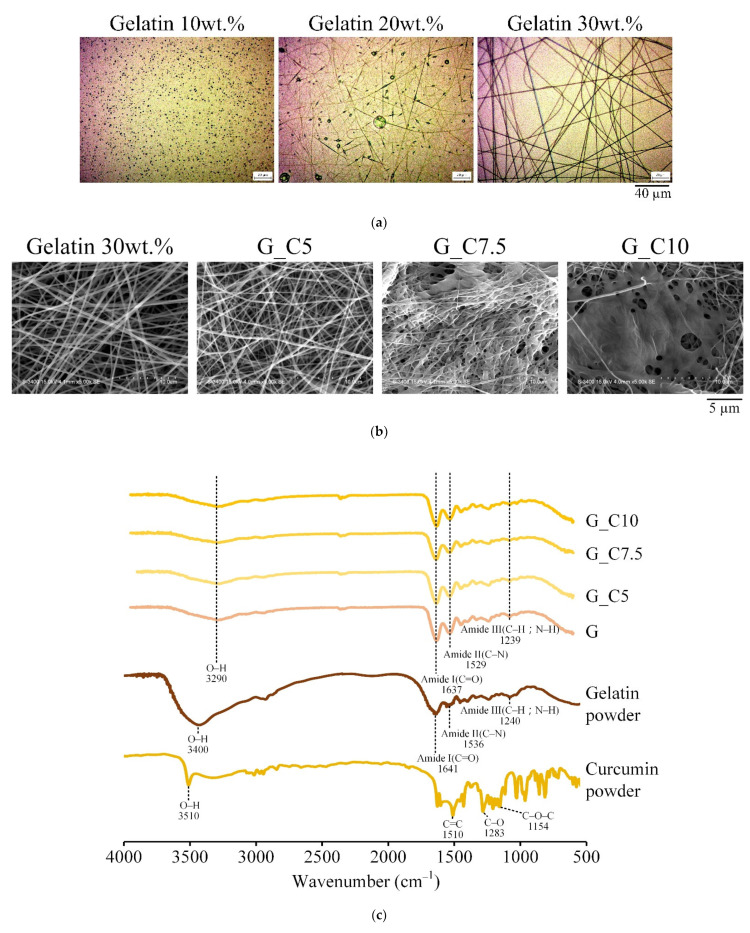
(**a**) OM images of electrospun membranes with different concentrations of gelatin; (**b**) SEM images and (**c**) IR spectra of electrospun gelatin membranes (G, G_C5, G_C7.5, and G_C10) with different concentrations of curcumin (0 wt.%, 5 wt.%, 7.5 wt.%, and 10 wt.%, respectively).

When the gelatin solution concentration was 10 wt.%, the collected polymers were in the form of beads (Figure 1a). When the gelatin concentration was 20 wt.%, the polymer transformed slightly into continuous filaments but still had attached beads. The gelatin concentration was increased to 30 wt.%, and the electrospun polymers became smooth filaments without beads; thus, the gelatin concentration of 30 wt.% was used for the subsequent experiments. Figure 1b shows the fiber morphology and microstructure observed on the surface of gelatin membranes loaded with different concentrations of curcumin. The OM results could be verified from the images. The gelatin group without curcumin (G) showed a smooth filament with no droplet generation, and the gelatin G_C5 added with 5 wt.% curcumin showed better fiber morphology. When G_C7.5 and G_C10 were added to the 7.5 wt.% and 10 wt.% groups, respectively, a large area of lumps appeared on the filament. Accordingly, the G_C5 group was selected for the subsequent tests.

The infrared spectrum of curcumin-loaded gelatin showed that the characteristic peak wavelength of gelatin is the absorption bands of amide I (C=O; C–N) at 1641 cm^−1^ (Figure 1c), amide II (N–H) at 1536 cm^−1^, and amide III (C=N) at 1240 cm^−1^ [20]. The characteristic peak wavelength of curcumin showed that the absorption band of C=C is at 1510 cm^−1^, the band of aromatic C–O is at 1283 cm^−1^, and the band of C–O–C is at 1154 cm^−1^. Comparison of the G spectra without curcumin and G_C5, G_C7.5, and G_C10 containing curcumin revealed that the curcumin-loaded gelatin membrane had no obvious curcumin characteristic peaks, similar to the literature results [21].

### 3.2. Outer Layer Selection

**Figure 2:**
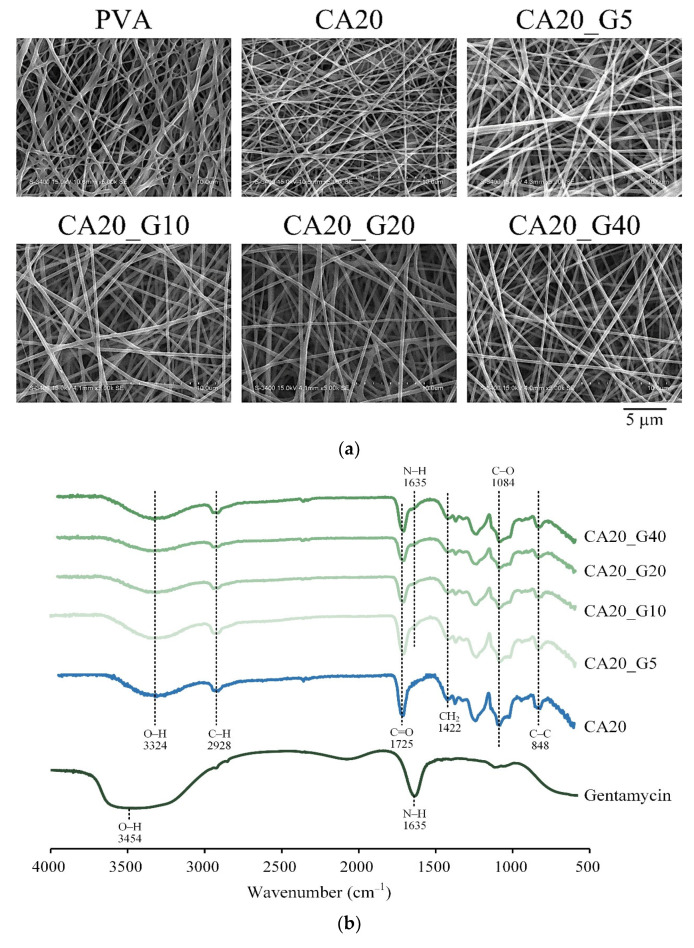
(**a**) SEM microstructural images and (**b**) IR spectra of 20 wt.% citric-acid-crosslinked PVA membranes (CA20, CA20_G5, CA20_G10, CA20_G20, and CA20_G40) with different concentrations of gentamicin (0%, 5%, 10%, 20%, and 40% by weight, respectively).

Observation of the fiber morphology and structure of CA-crosslinked PVA membranes with different gentamicin concentrations (Figure 2a) demonstrated that all the gentamicin-loaded groups had the same fiber morphology as CA20 without gentamicin. The results indicated that adding gentamicin did not alter the fiber morphology of CA20 in this study.

Figure 2b shows the infrared spectra of CA20 and gentamicin. The characteristic peak wavelength of gentamicin is the absorption band of O–H at 3435 cm^−1^, and the band at 1635 cm^−1^ is N–H [22]. Adding different proportions of gentamicin in CA20_G5, CA20_G10, CA20_G20, and CA20_G40 did not affect the main characteristic peak of CA20, but the absorption peak of gentamicin N–H appeared at 1635 cm^−1^, which proved that gentamicin was indeed loaded onto the PVA membrane [22].

**Figure 3:**
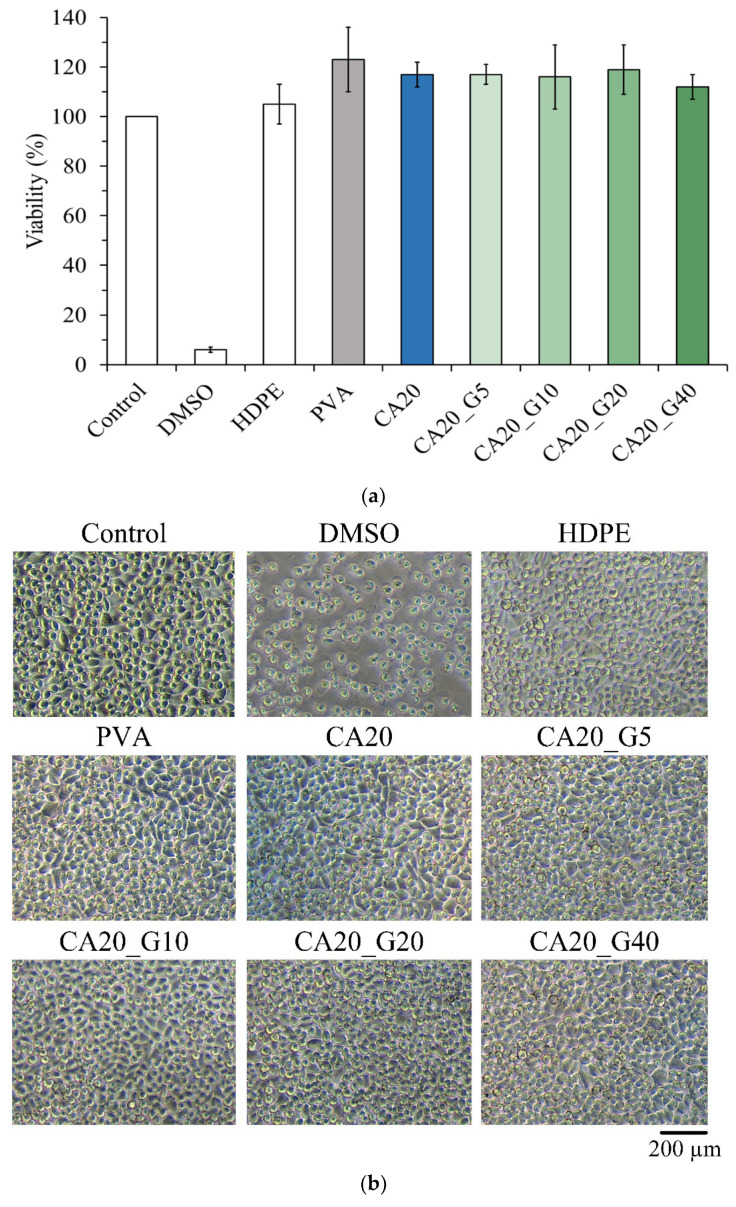
Viability of L929 cells cultured for 1 day in citric-acid-crosslinked PVA membrane extracts containing different concentrations of gentamicin: (**a**) quantitative test (*n* = 6) and (**b**) qualitative observation.

According to ISO 10993-5, if the survival rate of cells in each group is higher than 70% compared with the control after culturing with the extract for 1 day, the sample has no cytotoxicity. In this study, extracts of CA-linked PVA were incubated with L929 cells for 1 day for quantitative cytotoxicity. The results showed that all concentrations of gentamicin loaded to CA20 were not cytotoxic. In Figure 3b, the cell morphology of each group was similar to that of the control without denaturation, indicating that the samples did not cause cell degeneration or death.

**Figure 4:**
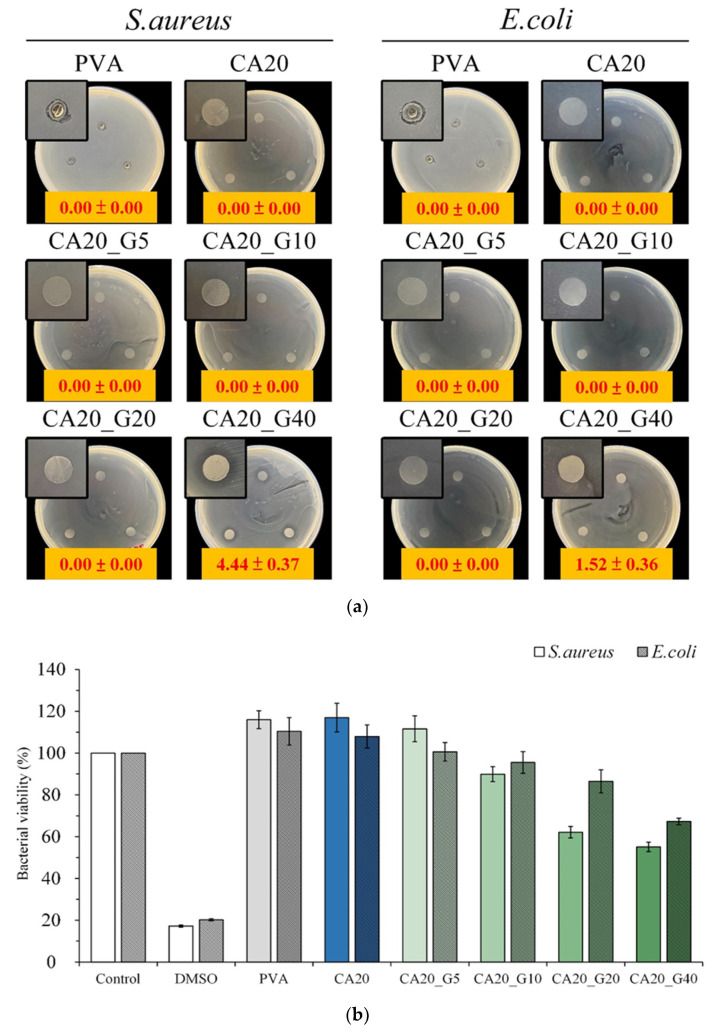
Antibacterial effect of different concentrations of gentamicin in citric-acid-crosslinked PVA membrane on *S. aureus* and *E. coli* cultured for 1 day: (**a**) qualitative inhibition (area unit: mm) and (**b**) quantitative test (*n* = 3).

The antibacterial activity of *Staphylococcus aureus* and *Escherichia coli* cultured for 1 day was qualitatively determined from different loaded concentrations of gentamicin in CA-linked PVA membranes, and only CA20_G40 had an obvious antibacterial zone (Figure 4a). This finding showed that the antibiotic concentration of the CA20_G40 group had a remarkable antibacterial effect. Further comparison of the quantitative test in Figure 4b revealed that the bacterial survival rates of CA20_G20 and CA20_G40 were lower than those of the control, indicating that gentamicin had an antibacterial effect at this concentration. This finding confirmed that CA20_G40 was more resistant to bacteria than CA20_G20; thus, it was selected for the outer layer of the multilayer membrane.

### 3.3. Sandwich PVA/Gelatin/PVA Membrane Characterizations

**Figure 5:**
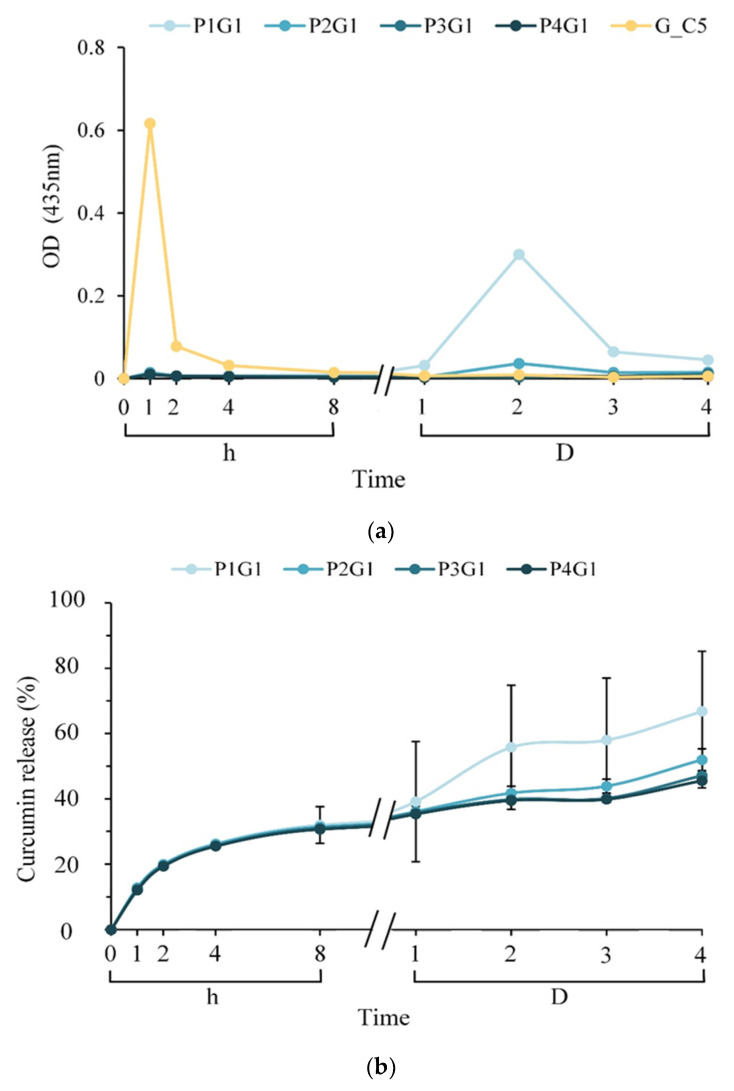
Curcumin (**a**) release amount at each time point and (**b**) cumulative release rate (*n* = 5) of curcumin after soaking in PBS for different times of the multilayer membrane under different process conditions. That is, the bifacial spinning times for each side were set to 1, 2, 3, and 4 h to obtain P1G1, P2G1, P3G1, and P4G1 groups, respectively.

Figure 5a shows the amount of curcumin released at each time point after using gelatin in the inner layer of the sandwich; the outer layer of the sandwich was electrospun with PVA on both sides of the inner layer to form a sandwich membrane soaked in PBS for different time points. After soaking in PBS for 1 h, the release of curcumin from G_C5 was significantly higher than that from other groups. This sudden release may be caused by the swelling of the structure and sliding release of curcumin from gelatin and PVA hydrogels. Although the multilayer membranes of P1G1, P2G1, P3G1, and P4G1 also showed rising release curves after soaking for 1 h, the curcumin amount was much lower than that of G_C5, presumably because the gelatin layer in the middle increased the pathways for curcumin to diffuse through the outer layers. The permeability between the PVA outer layer and the antibiotic layer also plays an important role in inhibiting the release of curcumin. On day 2 of immersion, when the P1G1 sandwich structure was deprived, the outer two PVA layers separated from the middle layer in PBS, resulting in increased release.

**Figure 6:**
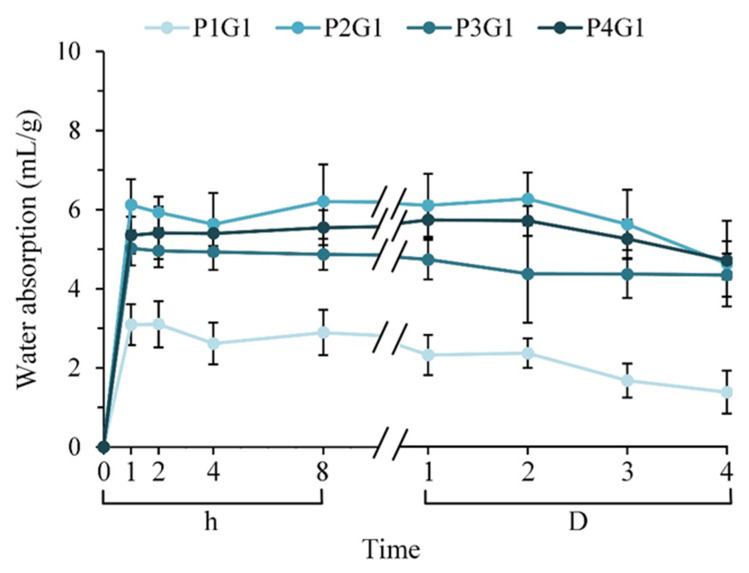
Water absorption of curcumin-impregnated multilayer membranes soaked in PBS for 4 days under different process conditions (*n* = 12).

Figure 6 shows the water absorption of different groups of sandwich PVA/gelatin/PVA structure multilayer membranes soaked in PBS at different times. The water content of each group rose sharply within 1 h of immersion and then reached a plateau until day 4 of immersion. A comparison of the water absorption of each group demonstrated that P1G1 could absorb about 3 mL of liquid per gram of membrane, and P2G1, P3G1, and P4G1 could absorb about 5–6 mL of liquid per gram of membrane. The relative water content of P1G1 was relatively low, presumably due to the short spinning time of the outer layer and the obvious reduction in the intersecting pores of the filaments. However, the water absorption did not increase with increasing electrospinning time of the PVA outer layer, which may be due to the competition between water cohesion and water adhesion within the PVA filaments.

**Figure 7:**
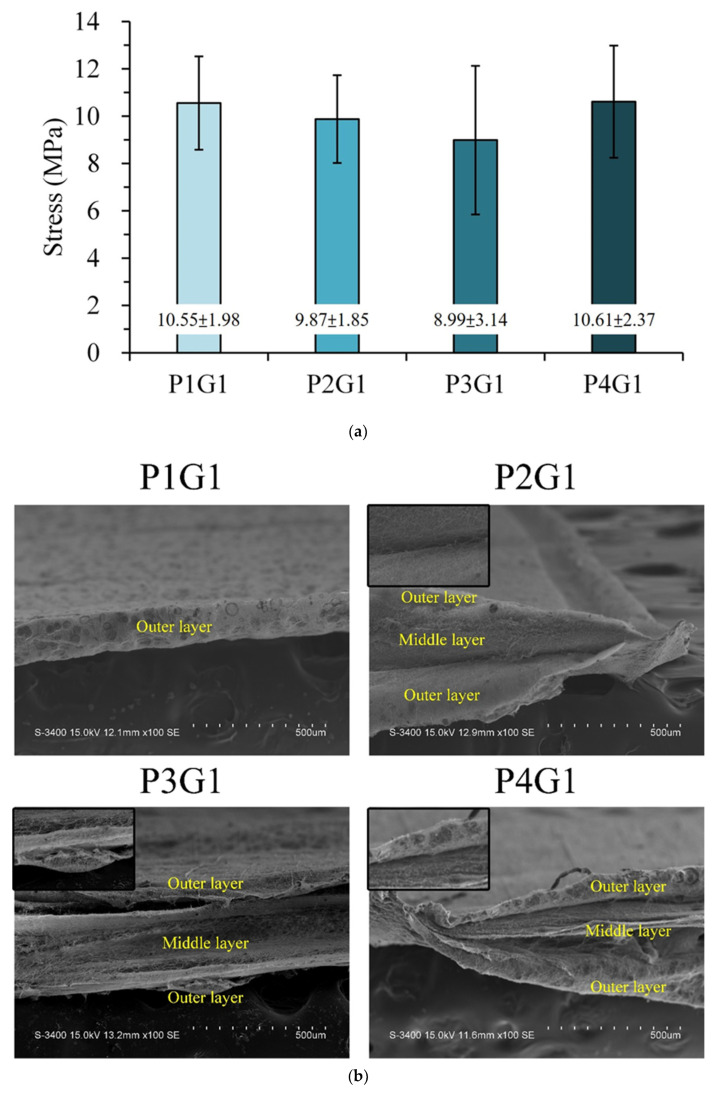
(**a**) Tensile strengths of sandwich PVA/gelatin/PVA structures with double-sided spinning time on each side set to 1, 2, 3, and 4 h to obtain P1G1, P2G1, P3G1, and P4G1 groups, respectively. All groups after one-way ANOVA analysis were shown to be nonsignificant (*p* > 0.05). (**b**) Cross-sectional images of fractured sandwich structures with double-sided spinning time on each side set to 1, 2, 3, and 4 h to obtain P1G1, P2G1, P3G1, and P4G1 groups, respectively.

Figure 7a shows the tensile strength of the multilayer membranes at different treatment times. However, no significant difference was found in the tensile strength of each group, indicating that the outer PVA layers on both sides of the sandwich PVA/gelatin/PVA membrane were not the main contributors to the loading. Figure 7b shows the SEM image of the cross-sectional fracture of the multilayer membrane under different conditions. The fracture section had an obvious three-layer sandwich PVA/gelatin/PVA structure, whereas the sandwich layer structure in P1G1 was less obvious, which is consistent with the previous inference. The tensile test results showed that the adhesion between the outer PVA layer and the middle crosslinked gelatin layer resulted in the inability to transmit force to the outer two layers to produce a composite additive effect. Therefore, changing the spinning time of the outer membrane did not affect the tensile strength.

### 3.4. Antibacterial Activity of Sandwich PVA/Gelatin/PVA Membrane

**Figure 8:**
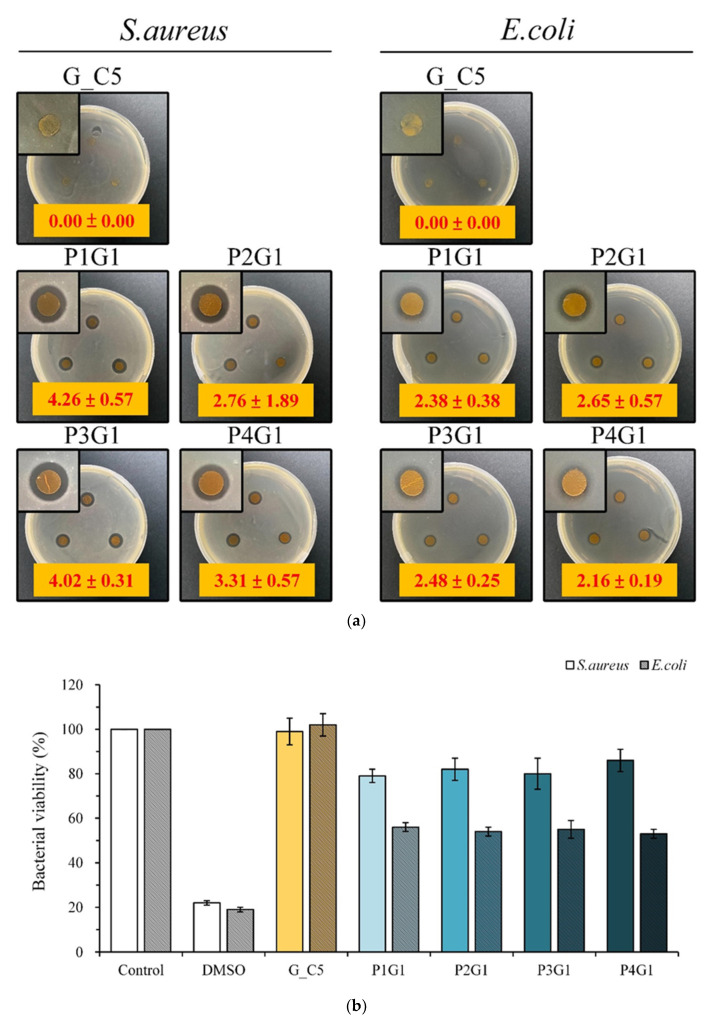
Antibacterial effect of multilayer membrane on *S. aureus* and *E.coli* in contact culture for 1 day under different process conditions: (**a**) qualitative (unit: mm) and (**b**) quantitative test (*n* = 3).

The qualitative antibacterial tests of different multilayer groups against *S. aureus* and *E. coli* cultured for 1 day are shown in Figure 8a. G_C5 had no antibacterial band against *S. aureus* and *E. coli.* Therefore, curcumin itself had no antimicrobial activity against *S. aureus* and *E. coli*, at least at this concentration. The antimicrobial effect released by gentamicin was not synergistically antimicrobial with that released by curcumin because no significant difference was found in the size of the antimicrobial zone in the multilayer membrane group.

Figure 8b shows the quantitative test of the antibacterial effect of different multilayer membrane groups on bacteria cultured for 1 day. The bacterial viability of G_C5 was higher than that of the control, indicating that G_C5 had no antibacterial effect. However, the viability of the bacteria was lower than that of the control, confirming that P1G1, P2G1, P3G1, and P4G1 had antibacterial ability. All the sandwich structural groups released gentamicin, and the antibacterial activity against *E. coli* was greater than that against *S. aureus.*

### 3.5. In Vitro Interaction of L929 Cells with Sandwich PVA/Gelatin/PVA Membranes

**Figure 9:**
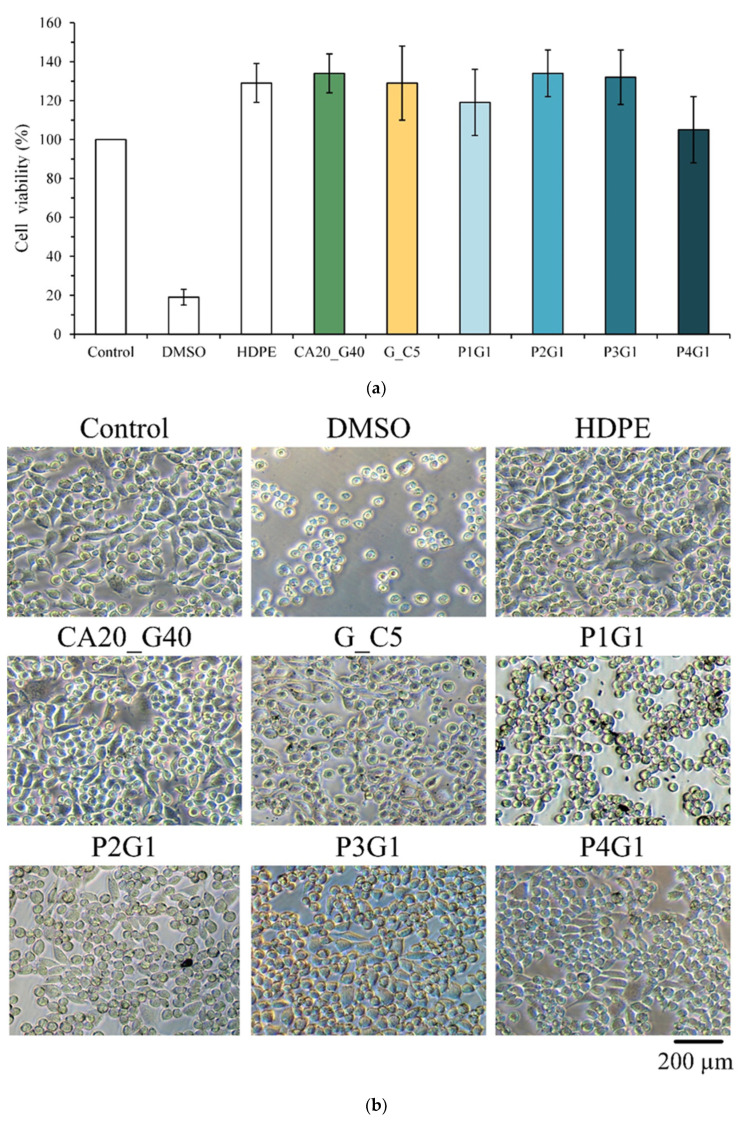
Cytotoxicity of (**a**) quantitative (*n* = 6) and (**b**) qualitative tests of multilayer membrane extracts cultured with L929 cells for 1 day.

After the extract was cultured with L929 cells for 1 day, the survival rate of cells in each group was higher than 70% (Figure 9a), indicating that the multilayer membrane group had no cytotoxicity. The qualitative observation of the experimental groups showed that the cell morphology of each group was similar to that of the control (Figure 9b). However, compared with the control, the P1G1 cells showed a slightly spherical cell shape, which was speculated to be due to the endocytosis of P1G1 dispersed fibers, resulting in degeneration and death.

**Figure 10:**
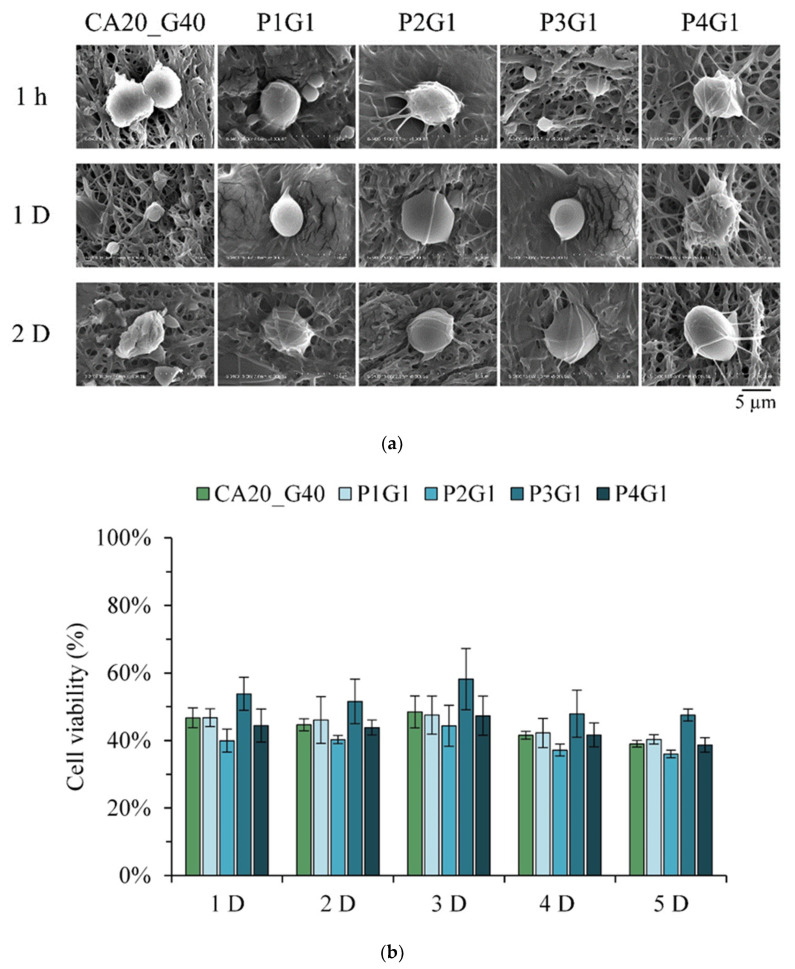
(**a**) Initial contact of cells was observed within 2 days after multilayer membranes under different process conditions were attached to L929 cells. (**b**) Cell proliferation test within 5 days of contact culture between multilayer membrane and L929 cells under different process conditions (*n* = 6).

Figure 10a shows the SEM images of L929 cells attached to different multilayer membrane groups at different times. After the cells were cultured for 1 h, spherical morphologies of the cells were attached to the fibers. At days 1 and 2 of the culture, the cells in each group did not change, and they were still spherical, indicating that the adhesion effect was not good. Figure 10b shows the cell proliferation of different groups and L929 cell contact culture for different times. Even when the L929 cells were cultured in contact with the sandwich samples until day 5, the cell viability of each group did not increase over time. These results showed that the sandwich membrane has the ability to resist cell adhesion and help prevent secondary damage during wound replacement [23].

## 4. Discussion

Hydrogels are one of the most dynamic materials because of their loose and porous three-dimensional network structure, biodegradability, hydrophilicity, easy grafting with other bioactive materials, and application in different forms [24]. Physiologically, the wound healing process is a distinguishable cascade involving hemostasis, anti-inflammation, cell proliferation, angiogenesis, and tissue remodeling at the site of skin injury [25]. Wounds that fail to heal within a foreseeable time frame will lead to further complications such as poor healing and infection and, eventually, chronic wounds [26]. Drug-loaded electrospun hydrogel membranes can overcome the limitations of traditional wound dressings, thereby providing sustained drug release to protect the wound surface for longer periods [27,28,29]. Modern wound dressings have evolved to be multifunctional, not only creating a moist environment, which is important for re-epithelialization, but also controlling fluid balance through a porous structure, thereby avoiding dehydration of the wound bed and accumulation of exudate, thus promoting wound healing [30,31,32]. Thus, the properties created by the multilayer membrane can maintain a moist environment and liquid absorbency, handle wound exudate, allow gas exchange, ensure good hemostasis, passively prevent wound infection or active antimicrobial control of wound odor, and provide sufficient strength to prevent cell adhesion and normal cell proliferation and migration to facilitate the healing process [33,34]. In addition, multilayer membranes used as dressing medical devices are required to be non-cytotoxic, non-fibroblast-adherent, and non-sensitizing [35,36]. The initial release of gentamicin from the double-sided outer layer shows that the electrospun sandwich structure has higher encapsulation efficiency, initial burst release to obtain antibacterial activity, and the ability to maintain biocompatibility (Figure 8 and Figure 9). In addition, in terms of cell adhesion (Figure 10), the sandwich membrane has the ability to resist cell adhesion, which helps to prevent secondary damage during wound replacement. From the perspective of humanization, the design of an ideal wound dressing should also ensure clinical compliance, such as convenient application, painless removal, low dressing replacement frequency, and high-cost performance [37]. Compared with other conventional fiber wound dressings, the nanofiber hydrogel electrospun multilayer membrane has a very high specific surface area, and the high pore aggregation area composed of nanofiber intersections results in small pore size and good air permeability, which could block exogenous bacteria from entering the wound site [38]. The above capabilities depend on the polymer solution, the electrospun fiber diameter, and the thickness of the fiber layer [39]. Researchers have investigated multilayer nanofibrous structures consisting of an intermediate layer with active ingredients/drugs and covered layer by layer with different fiber membranes. They found that the initial burst release may be reduced, with a slower release achieved with a thicker outer cover [38,39,40,41]. Accordingly, in the sandwich structure of multilayer membranes, the diffusion barrier through the outer layer could delay and sustain the release of the drug carried from the middle layer. Hydrogel-based electrospun membranes are considered to be an easy and effective strategy to fabricate ECM-like nanofiber structures [18]. Similarly, researchers investigated electrospun PVA/sodium alginate hydrogel composite nanofiber transdermal patches loaded with ciprofloxacin [41]. They found that the healing properties through in vivo studies indicated that the drug-loaded PVA/sodium alginate composite nanofibers had the best performance. Curcumin, a natural polyphenol, can significantly improve symptoms in patients with colitis, and many studies have shown that curcumin has excellent anti-inflammatory, antioxidant, and antibacterial properties [42,43,44]. However, the in vivo bioavailability of curcumin is low due to relatively low absorption from the small intestine coupled with extensive reductive and conjugative metabolism in the liver and elimination through the gallbladder. In recent years, the incorporation of curcumin into hydrogels for the treatment of inflammatory bowel disease has eliminated these drawbacks [24]. In addition, it was found that curcumin encapsulated in chitosan/sodium alginate hydrogel enhanced the secretion of pro-inflammatory cytokines to inhibit macrophages, and the release of curcumin through nanocarriers had the best therapeutic effect [45]. In the present study, due to the high loading capacity and uniform release characteristics of electrospinning, antibiotics were placed on the outer layer of PVA fibers, and curcumin was loaded on the middle layer of gelatin fibers without destroying the strength and biocompatibility of the composite membrane. Meanwhile, for drug delivery systems, the sandwich nanofibrous structure has the advantage of the sustained release of multiple carriers.

## 5. Conclusions

In this study, a gentamicin-loaded CA-crosslinked PVA membrane as the outer layer on both sides and a curcumin-loaded gelatin membrane as the middle layer was successfully composited into a functional multilayer membrane by electrospinning. The sandwich PVA/gelatin/PVA structure could absorb five to six times more PBS per gram, which would help to absorb wound exudate. The gentamicin contained in PVA had an antibacterial effect and could prevent wound infection. Compared with the other groups, P1G1 had poor performance in terms of release, water absorption, and cytotoxicity due to the shorter spinning time of the outer layer, whereas no significant difference was found in the performance of P2G1, P3G1, and P4G1. The surface of the multilayer membrane was hydrophilic, but it reduced the adhesion and proliferation of fibroblasts, and it could reduce secondary damage to the wound when used in artificial wound dressings. The results showed that the spinning time of the outer layer of the multilayer membrane should be more than 2 h to achieve the expected composite and function. The sandwich structure in the multifunctional multilayer membrane developed in this study could be practically applied to wound dressings in the future to reduce the risk of bacterial infection and accelerate wound repair.

## Figures and Tables

**Table 1:** Nomenclature of designated gelatin membranes with different concentrations of curcumin.

Designated Groups	Gelatin ^a^ (wt.%)	Curcumin (% by Weight of Gelatin)
G	30	0
G_C5	30	5.0
G_C7.5	30	7.5
G_C10	30	10.0

^a^ 30 wt.% of gelatin was relative to the electrospinning solution without curcumin, that is, 10 mL of 40 vol.% acetic acid with a specific gravity of 1.05 g/mL was 10.5 g, and the gelatin added was 4.37 g. Therefore, the gelatin in the solution was 30% by weight.

**Table 2:** Nomenclature of citric acid cross-linked polyvinyl alcohol membranes with different concentrations of gentamicin.

Designated Groups	PVA ^b^ (wt.%)	Citric Acid (% by Weight of PVA)	Gentamycin (mg/10 mL Spinning Solution)
CA20_G5	8	20	5
CA20_G10	8	20	10
CA20_G20	8	20	20
CA20_G40	8	20	40

^b^ The 8 wt.% of PVA was relative to the electrospinning solutions without citric acid and gentamycin, that is, 8.7 g of PVA was dissolved in 100 mL of water, and the PVA in the aqueous solution was 8% by weight.

**Table 3:** The nomenclature of the PVA-gelatin-PVA sandwich structure determined by the different spinning times of the PVA double-sided outer layers.

Designated Membrane with a Sandwich Structure of PVA-Gelatin-PVA	Gelatin Double-Sided Outer Layer PVA Spinning Time (h)	Middle Layer Gelatin Spinning Time (h)
P1G1	1	1
P2G1	2	1
P3G1	3	1
P4G1	4	1

## Data Availability

The data presented in this study are available on request from the corresponding author.
